# Plasma Proteins Invariant to Diet in Celiac Disease: Results From a Proteomics Study on the UK Biobank

**DOI:** 10.1016/j.gastha.2025.100803

**Published:** 2025-09-12

**Authors:** Isabel A. Hujoel, Po-Ru Loh, Margaux L.A. Hujoel

**Affiliations:** 1 Division of Gastroenterology, University of Washington, Seattle, Washington; 2 Brigham and Women’s Hospital and Harvard Medical School, Boston, Massachusetts; 3 Program in Medical and Population Genetics, Broad Institute of MIT and Harvard, Cambridge, Massachusetts

**Keywords:** Gluten challenge, Celiac disease, Proteomics, Nonceliac gluten sensitivity, Gluten-free diet

## Abstract

**Background and aims:**

Our serologic and pathologic markers of celiac disease normalize with a gluten-free diet. Identifying celiac disease in those who are already on a gluten-free diet is therefore difficult and currently requires a gluten challenge. The gluten challenge may have low sensitivity and is often not palatable to patients due to associated morbidity from gluten ingestion. We aimed to identify potential serologic markers of celiac disease invariant to diet by performing proteomic analysis.

**Methods:**

We performed a proteomic analysis using the UK Biobank, specifically looking for plasma protein levels associated with celiac disease among individuals self-reporting a gluten-free diet. Celiac disease was identified through the ICD-10 code of K90.0. We limited our analysis to those diagnosed with celiac disease prior to proteomic data being collected. We inverse-rank normalized proteomic measurements. Our primary analysis was looking at differential expression between those diagnosed with celiac disease who reported being gluten-free and controls.

**Results:**

A total of 1044 individuals received a diagnosis of celiac disease prior to proteomic analysis. Of these individuals, 141 were administered a dietary questionnaire, and 132 reported being gluten-free. There were 4 proteins which were significantly higher in this group, however only two were unique to celiac disease: carboxypeptidase A2 and integrin subunit beta 7.

**Conclusion:**

Although further diagnostic accuracy studies are required, this study identified 2 potential markers for celiac disease that are invariant to diet and commercially available. This has dramatic implications for clinical practice.

## Introduction

Celiac disease is a common immune-mediated disorder that is triggered by ingestion of gluten and leads to an enteropathy. Diagnosis in adults currently requires abnormal duodenal pathology and, in most cases, abnormal serologic testing (with the exception of seronegative celiac disease).^1^ As individuals with celiac disease go gluten-free, the pathologic and serologic tests will normalize to the point where someone with celiac disease on a gluten-free diet cannot be distinguished from someone without celiac disease.^2^^,^^3^ This normalization creates a diagnostic challenge when someone has already adopted a gluten-free diet prior to seeking a formal diagnosis of celiac disease. Receiving a diagnosis of celiac disease can potentially dramatically impact quality of life by determining how strict gluten avoidance should be.^4^ In nonceliac gluten sensitivity, symptoms guide decisions on gluten exposure, and cross-contamination may be less of a concern.^4^ In celiac disease, gluten avoidance is much stricter. A formal diagnosis also has implications for family screening, given the genetic nature, as well as long term monitoring and care.^4^

The gluten-free diet is increasing in popularity.^5^ Nearly one-third of the US population report avoiding gluten, the majority of who do not carry a diagnosis of celiac disease.^6^ Currently, there are no markers available in the clinical setting to identify celiac disease that are invariant to diet, and instead genetic testing—specifically related to the human leukocyte antigen genes—followed by a gluten challenge is the only option. Although the exact details are controversial, a gluten challenge involves significant gluten consumption for a period of several weeks.^7^^,^^8^ Studies suggest the gluten challenge may have low sensitivity for identifying celiac disease, and additionally, the gluten challenge is not feasible for many due to the associated symptoms triggered from gluten ingestion.^9^^,^^10^ We therefore aimed to identify potential serologic markers of celiac disease that are invariant to diet by performing proteomic analysis on data from the UK biobank.

## Methods

We used data from the UK Biobank for this study.^11^ The UK Biobank includes prospective data on 500,000 UK residents (ages 40–69). These data cover imaging, genetics (including whole genome sequencing), lifestyle, biomarkers, questionnaires, and fluid samples. Recruitment began in 2006.

Inpatient medical conditions are identified in the UK Biobank with International Classification of Diseases (ICD)-9, ICD-10, Office of Population Censuses and Surveys-3 and Office of Population Censuses and Surveys-4 coding. We extracted the date of first inpatient diagnosis for celiac disease (ICD-10 code K90.0; Field 41,280), and the date when Crohn's disease (K50; Field 131,626), ulcerative colitis (K51; Field 131,628), or other noninfective gastroenteritis and colitis (K52; Field 131,630) were first reported. We determined if, based on an administered questionnaire, the individual had reported following a special type of diet (Field 20,086).

Plasma proteomic data were recently made available for 54,219.

UK Biobank individuals.^12^ Two-thousand nine-hundred and twenty-three unique proteins (2941 protein analytes) were measured within the blood plasma samples from this subset of individuals using the antibody-based Olink Explore 3072 Proximity Extension Assay. We categorized an individual as having celiac disease before assessment if their first inpatient diagnosis for celiac disease was earlier than their date of attending the assessment center (Field 53).

We restricted to a set of unrelated individuals with plasma proteomic data available and inverse-rank normalized proteomic measurements; this transformation was done per protein. In linear association models we included the following covariates: age, age,^2^ sex, age × sex, age^2^ × sex, batch, UK Biobank center, UK Biobank genetic array, genetic ancestry, time between blood sampling and measurement and the first 20 genetic principal components.

Our primary analysis was testing for differential expression among those diagnosed with celiac disease prior to assessment who reported being gluten-free (n = 19) to those with no diagnosis of celiac disease (n = 52,262). We tested all proteins (n = 2911) which had at least 10 measurements for both cases (reported gluten-free individuals diagnosed with celiac disease prior to blood sampling; n = 19) and controls (individuals not diagnosed with celiac disease; n = 51,765; Figure). Secondary analyses compared those diagnosed with celiac disease prior to assessment who did not complete a diet questionnaire (n = 98) to those with no diagnosis of celiac disease, comparing those diagnosed with inflammatory bowel disease or other noninfective gastroenteritis and colitis (n = 4049 for a diagnosis at any point and 2029 diagnosed before assessment) to those not diagnosed, and comparing individuals who reported being gluten-free (n = 141) to those not reporting to be gluten-free (n = 6618) among those with no diagnosis of either inflammatory bowel disease or celiac disease. All secondary analyses were restricted to the proteins found to be significantly different between cases and controls in our primary analysis.

**Figure:**
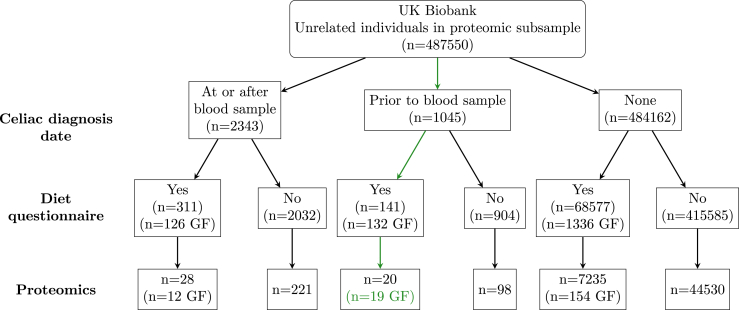
This diagram shows the sample sizes available in UK Biobank meeting various data requirements (eg, having proteomic data available). The initial set of considered individuals consists of the entire UK Biobank removing one individual from pairs who are related (kinship > .0884) who both have proteomic data available. GF = gluten-free.

## Results

Within the UK Biobank (n = 487,550), 3388 individuals had an inpatient diagnosis for celiac disease, 1045 received their first inpatient diagnosis prior to attending the assessment center and 2343 after. Of all participants in the UK Biobank, 53,020 had plasma proteomic data available. Among the individuals with a diagnosis of celiac disease who were given a diet survey (n = 452), 94% reported to be gluten-free among those diagnosed prior to assessment (132 reported gluten-free while 9 did not) and 41% reported to be gluten-free among those diagnosed after assessment (126 reported to be gluten-free while 185 did not). See Figure for additional information regarding sample sizes.

There were 4 proteins at significantly (Bonferroni-significant) higher levels among those diagnosed with celiac disease who reported to be gluten-free compared to those not diagnosed with celiac disease. These included anterior gradient 2, carboxypeptidase A2 (CPA2), integrin subunit beta 7 (ITGB7), and POF1B actin-binding protein (POF1B) (Table).

**Table:** Effect Sizes and Significance for 4 Inverse-Normalized Protein Levels—AGR2, CPA2, ITGB7, POF1B—Among Various Subgroups (Compared to all Controls) Is Shown

Protein	Celiac disease on a gluten-free diet		Celiac disease and no diet survey		Inflammatory bowel disease		Gluten-free controls
	Beta (SE)	*P*	Beta (SE)	*P*	Diagnosis at any time	Diagnosed prior to assessment	*P*
AGR2	1.10 (0.22)	9.7e-07	0.98 (0.10)	1.6e-21	2.2e-21	1.8e-15	.58
CPA2	0.99 (0.23)	1.1e-05	1.25 (0.10)	4.2e-34	0.36	0.96	.97
ITGB7	1.00 (0.23)	1.0e-05	1.27 (0.10)	1.8e-35	0.04	0.50	.17
POF1B	1.15 (0.25)	2.9e-06	1.02 (0.11)	5.6e-20	9.4e-11	9.7e-10	.11

AGR2, anterior gradient 2; SE, standard error.

These were the 4 proteins found to be significantly associated with celiac disease on a gluten-free diet. Associations were computed using a linear regression and are adjusted for age, age,^2^ sex, age × sex, age^2^ × sex, batch, UKB center, UKB genetic array, genetic ancestry, time between blood sampling and measurement and the first 20 genetic principal component.

These associations were similar in magnitude, but statistically stronger, among those diagnosed with celiac disease prior to assessment who did not complete a diet questionnaire. As 95% (19 of 20) of those with celiac disease given a diet survey reported being gluten-free, it is reasonable to assume a similar proportion of those with celiac disease and not given a diet survey were gluten-free and thus the similar magnitude and statistically stronger associations in this subset can be expected due to the increase in power (number of cases is 19 vs 98; Figure). While anterior gradient 2 and POF1B were also associated with inflammatory bowel disease or other noninfective gastroenteritis and colitis, CPA2 and ITGB7 were uniquely significantly associated with celiac disease. None of these 4 proteins were significantly associated with gluten-free diet among controls.

## Discussion

Through plasma proteomic analysis on data from the UK Biobank we identified 4 proteins that were significantly associated with celiac disease, despite individuals reporting following a gluten-free diet. Two of these proteins, CPA2 and ITGB7, were unique to celiac disease. These proteins may provide an alternative to a gluten challenge in identifying celiac disease in those already on a gluten-free diet.

Our findings are consistent with a 2024 study which used the UK Biobank to perform plasma proteomic analysis for several diseases including celiac disease.^13^ They identified a specific protein signature for celiac disease involving 20 proteins, three being the transglutaminase 2, ITGB7, and CPA2. Another proteomic study done on both plasma (30 individuals with CeD compared to 30 healthy controls) and duodenal tissue (19 individuals with CeD compared to 19 healthy controls) did not identify CPA2 or ITGB7 in the plasma of those with celiac disease.^14^ This difference could be because this study specifically analyzed those who were newly diagnosed with celiac disease (so likely not on a gluten-free diet).^14^ This difference could also be due to a small sample size or the use of a different proteomic approach (plasma was analyzed using liquid chromatography–tandem mass spectrometry with the Four-Dimensional Data-Independent Aquisition technique). Unique to our study was further analysis on protein signature as related to being gluten-free or not.

Prior studies highlight possible biological explanations behind the association of these proteins, CPA2 and ITGB7, with celiac disease. ITGB7 is a protein that can pair with alpha subunits to form integrin receptors—specifically in conjunction with integrin alpha 4 it can create integrin alpha4beta7. Integrin alpha(4)beta(7) along with mucosal addressin cell adhesion molecule 1 plays a crucial role in leukocyte trafficking to the gut and in turn plays a significant role in inflammation in the intestine.^15^^,^^16^ Mucosal addressin cell adhesion molecule 1 duodenal expression has been found to be significantly higher in active celiac disease, and this pathway likely plays an important role in the mucosal damage seen in celiac disease.^17^ The role of these proteins has been highlighted in inflammatory bowel disease and they are even a target of treatments such as vedolizumab.^18^ Notably, despite this association with inflammatory bowel disease, peripheral ITGB7 was not associated with inflammatory bowel disease in our study. Given the role of ITGB7 in inflammation, elevated serum levels could potentially indicate ongoing disease activity. Future studies are needed to explore the association of this protein with different states of disease activity (ie time on a gluten-free diet, pathologic confirming of healing). CPA2, by contrast, is an enzyme secreted by the pancreas that cleaves C-terminal amino acids from peptides and proteins, with a preference for large and aromatic residues. Two peptides from the C-terminal region of gliadin have been identified as potentially immunodominant in those with celiac disease.^19^

While there are no clinically available alternatives to the gluten challenge, there are options showing promise in the research setting. The most promising opportunity is the HLA-DQ-gluten tetramer, which studies show can accurately identify celiac disease, even in the absence of gluten consumption.^20^ Unfortunately, this test is limited by not being commercially available, requiring a significant volume of blood to perform, and being labor intensive.^21^ Other potential options include gluten-specific T cells which can be found in the blood after a 3 day gluten challenge, and a cytokine rise, most notably in interleukin-2, with a single gluten exposure.^22^ This rise in interleukin-2 appears to be unique to those with celiac disease.^23^^,^^24^ CD4 T-cell clonotypes have been found to remain in the peripheral blood of those with celiac disease for decades. Currently there has been limited investigation into the accuracy of these tests.^25^ The proteins identified in our study are both commercially available tests, bypassing the technical limitations of HLA-DQ-gluten tetramer.

Our study has several strengths. We analyzed a broad array of proteins (n = 2913) and had the ability to assess the impact of diet on the association. Moreover, as UK Biobank is a large population cohort we could also assess whether proteins were uniquely associated with celiac disease or whether they also had association with other digestive diseases. Our study also has potential weaknesses. Our study used proteomic data available via the UK Biobank Pharma Proteomics Project which was measured with the antibody-based Olink platform in a subset of UK Biobank individuals, confirming these results in additional cohorts or using orthogonal methods remains a future direction.^12^ The gluten-free diet was identified through survey answers, and it is possible that participants may not have answered truthfully. Additionally, the diagnosis of celiac disease (as well as inflammatory bowel disease) was determined through ICD coding, leading to the possibility of misclassification. Finally, the number of individuals who reported being gluten-free and additionally had proteomics data available was limited (n = 19). As such, this study may be underpowered to detect small differences in normalized protein levels, however the differences needed for potential biomarkers to be potentially clinically relevant are large and as such this data still provides important insights.

Our results suggest that there are 2 potential commercially available markers for celiac disease that are invariant to diet. Further studies on the diagnostic accuracy of these 2 proteins, either individually or jointly, are necessary as the reliance on ICD coding and self-report nature of gluten-free adherence in UK Biobank make assessing the predictive accuracy of these proteins difficult. Diagnostic accuracy studies are at high risk of bias and a prior study has highlighted this in relation to serologic testing for celiac disease and the dramatic impact these introduced biases can have on results.^26^^,^^27^ Future studies should therefore be carefully designed and adhere to the Standards for Reporting of Diagnostic Accuracy Studies guidelines.
